# vulcanSpot: a tool to prioritize therapeutic vulnerabilities in cancer

**DOI:** 10.1093/bioinformatics/btz465

**Published:** 2019-06-07

**Authors:** Javier Perales-Patón, Tomás Di Domenico, Coral Fustero-Torre, Elena Piñeiro-Yáñez, Carlos Carretero-Puche, Héctor Tejero, Alfonso Valencia, Gonzalo Gómez-López, Fátima Al-Shahrour

**Affiliations:** 1 Bioinformatics Unit, Spanish National Cancer Research Centre (CNIO), Madrid 28029, Spain; 2 Computational Biology Life Sciences Group, Barcelona Supercomputing Centre, Barcelona, Spain

## Abstract

**Motivation:**

Genetic alterations lead to tumor progression and cell survival but also uncover cancer-specific vulnerabilities on gene dependencies that can be therapeutically exploited.

**Results:**

vulcanSpot is a novel computational approach implemented to expand the therapeutic options in cancer beyond known-driver genes unlocking alternative ways to target undruggable genes. The method integrates genome-wide information provided by massive screening experiments to detect genetic vulnerabilities associated to tumors. Then, vulcanSpot prioritizes drugs to target cancer-specific gene dependencies using a weighted scoring system based on well known drug-gene relationships and drug repositioning strategies.

**Availability and implementation:**

http://www.vulcanspot.org.

**Supplementary information:**

Supplementary data are available at *Bioinformatics* online.

## 1 Introduction

Tumor progression and cancer cell survival usually depends on acquired genetic alterations like Loss-of-Function (LoF) of tumor suppressor genes and Gain-of-Function (GoF) of oncogenes. Such genetic dependencies (GDs) may confer specific tumor vulnerabilities that have been proposed to be therapeutically exploited (i.e. synthetic lethality) enabling cancer cells to be targeted selectively (Brunen and Bernards, 2017). Massive gene LoF experiments by RNAi and CRISPR such as Cancer Dependency Map (DepMap, Tsherniak *et al.*, 2017) and drug screenings across cancer cell lines such as Cancer Cell Line Encyclopedia (CCLE) (Barretina *et al.*, 2012), Genomics of Drug Sensitivity in Cancer (GDSC, Iorio *et al.*, 2016) and Cancer Therapeutic Response Portal (CTRP, Seashore-Ludlow *et al.*, 2015) have been systematically performed seeking for GDs and novel biomarkers of drug response in cancer. These projects have encouraged the development of *in silico* drug prescription strategies to link genomic alterations or GDs to potential therapies (Bridgett *et al.*, 2017; Piñeiro-Yáñez *et al.*, 2018; Rubio-Perez *et al.*, 2015). Here we introduce vulcanSpot (VULnerable CANcer Spot), a novel webtool to expand and prioritize the cancer therapeutic options targeting tumor-specific GDs detected in user’s query.

## 2 Methods and features

**Fig. 1.**
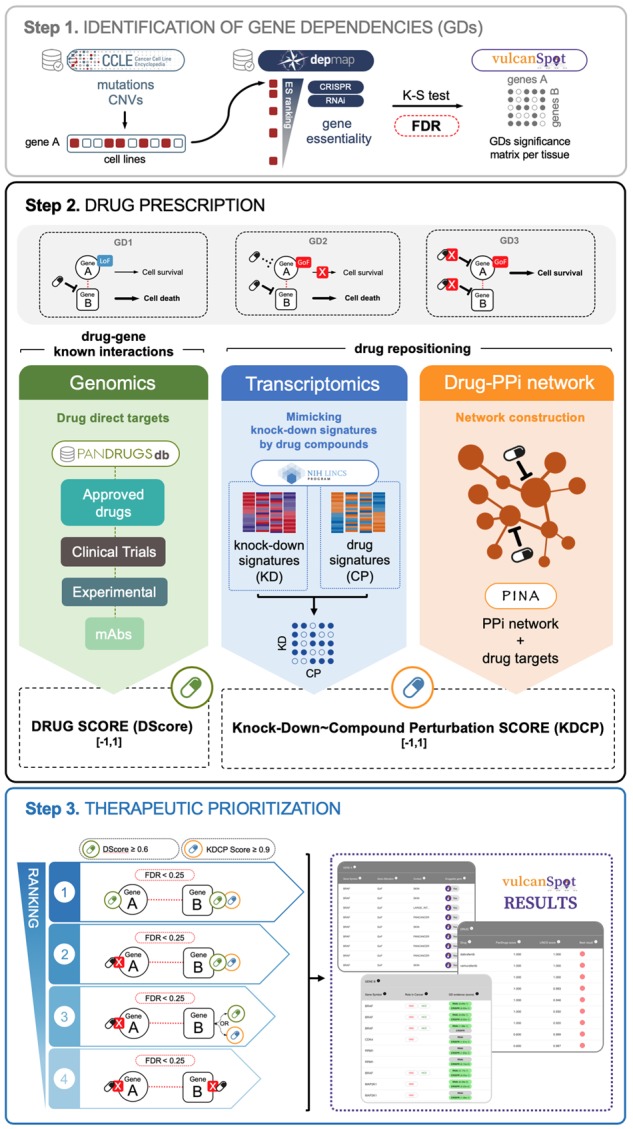
vulcanSpot workflow

vulcanSpot includes three main steps: (i) genome-wide identification of GDs considering distinct cellular contexts in cancer, (ii) *in silico* prescription of drugs that directly target those GDs or mimic the GDs depletion employing transcriptional signatures and protein–protein interaction (PPi) networks and (iii) therapeutic prioritization following the most relevant druggable associations (Fig. 1).

### 2.1 Identification of GDs in tumor-specific genotype contexts

vulcanSpot identifies GDs by integrating molecular profiles of DNA alterations and gene essentiality from the CCLE and DepMap datasets, respectively (Supplementary Table S1). To do this, genetic DNA alterations in protein-coding genes were classified into GoF and LoF based on their functional consequence (Futreal *et al.*, 2004). Then, cancer cell lines harboring a recurrent genetic alteration (Gene A) were interrogated using Kolmogorov-Smirnov test to evaluate whether there is a significant enrichment of such alteration across the ranked essentiality score (estimated by DepMap dependency score) for a given gene (Gene B) in all cell lines (Fig. 1). This statistical analysis detects significant GDs for PanCancer scenario or lineage-specific context, being gene A an altered input gene and gene B essential for cell viability upon this genotype context (Supplementary Materials S3 and Supplementary Fig. S1).

### 2.2 Drug prescription on vulnerable GDs

vulcanSpot distinguishes three types of vulnerable GDs (Fig. 1) depending on gene A and/or gene B druggability with current drugs (FDA approved and clinical trials).

*In silico* drug prescription is performed following two complementary approaches. First, vulcanSpot assesses the druggability of GDs using PanDrugs, a methodology to prioritize candidate drugs evaluating gene therapeutic actionability (Piñeiro-Yáñez *et al.*, 2018). Thus, vulcanSpot integrates: (i) PanDrugs database information about genes that can be directly targeted by a drug (direct targets) and (ii) PanDrugs drug score (DScore) to evaluate drug response and treatment suitability of each identified GD. Second, vulcanSpot extends the repertoire of suggested therapies targeting GDs using a drug repositioning approach. This method prioritizes those drugs whose transcriptional activity mimic the transcriptional change of a knocked-down gene of interest. To do so, we calculate the consensus gene expression signatures from a catalog of gene knock-down (KD) and compound perturbations (CP) expression profiles in cancer cell lines from the LINCS L1000 dataset (Subramanian *et al.*, 2017). A KDCP score representing the similarity between every pair of KD–CP signatures is calculated using the Total Enrichment Score (Iorio *et al.*, 2010). KDCP score also integrates PPi networks information (Cowley *et al.*, 2012). Distance between network nodes is employed to prioritize those drug compounds that act closer to the target of interest (see Supplementary Material S4.2).

### 2.3 Therapeutic prioritization

vulcanSpot final output offers a ranking of prioritized drugs to target statistically significant GDs (FDR < 0.25). The ranking is ordered taking into account DScore, KDCP score and the experimental validation of the proposed GD–drug pairs using drug sensitivity data from GDSC and CTRP (see Supplementary Materials S5–S8).

### 2.4 Implementation details

vulcanSpot back-end was written in JavaScript using the Node.js runtime environment, and it is supported by a PostgreSQL database to store the data in a relational model. The front-end is written in JavaScript using the React library for functional components, and the Material-UI library for the presentation layer. vulcanSpot also offers programmatic access through a RESTful API.

## 3 Conclusions

We have developed vulcanSpot, a novel genome-wide method to exploit massive screenings data to identify GDs and prescribe drugs using a combination of known drug-gene relationships and drug repositioning strategies. This methodology uncovers novel therapeutic strategies to target cancer-specific vulnerabilities. vulcanSpot and its full documentation are accessible at http://www.vulcanspot.org/.

## Funding

This work was supported by National Institute of Health Carlos III (ISCIII); Marie-Curie Career Integration Grant (CIG334361); and Paradifference Foundation. J.P.-P. is supported by Severo Ochoa FPI grant doctoral fellowship by the Spanish Ministry of Economy and Competitiveness. C.F.-T. is supported by Comunidad de Madrid [S2017/ 65 BMD-3778] (LINFOMAS-CM) co-financed by European Structural and Investment Fund.

*Conflict of Interest*: none declared.

## Supplementary Material

btz465_Supplementary_Data
