# Supplementary material for: vulcanSpot: a tool to prioritize therapeutic vulnerabilities in cancer
Source: Bioinformatics. 2019 Jun 7;35(22):4846–8. doi: 10.1093/bioinformatics/btz465 (PMC6853644; doi:10.1093/bioinformatics/btz465)

**Supplementary Materials for**

**“vulcanSpot: a tool to prioritize therapeutic vulnerabilities in cancer”**

Javier Perales-Patón, Tomás Di Domenico, Coral Fustero-Torre, Elena Piñeiro-Yáñez, Carlos Carretero-Puche, Héctor Tejero, Alfonso Valencia, Gonzalo Gómez-López and Fátima Al-Shahrour

# S1. Namespace for data integration

vulcanSpot integrates several resources comprehending individual molecular profiles of cancer cell lines (DNA alterations, basal gene expression, gene knock-down and drug-induced gene expression profiles, and gene essentiality), human protein-protein interactions and drug-gene associations (Suppl. Table 1). To address this integration, biological and chemical entities such as samples, compounds, genes and proteins are matched across resources using a common namespace. Standardized nomenclature for cell lines from the Cancer Cell Line Encyclopedia (CCLE, Broad Institute) was used to integrate individual molecular profiles for the same biological sample. Cancer cell lines were grouped by their primary tumour site based on meta information from the Cancer Cell Line Encyclopedia. Gene symbols (HUGO Gene Nomenclature Committee) were used as a common space to match the original gene and protein identifiers using *biomaRt* (R package v.2.34.2, GRCh37 feb2014 human assembly, ensEMBL mart). PubChem was used to match cases of disambiguation of compounds with different identifiers but corresponding to the identical chemical compound via API RestFul Service using the following order of preference: identical drug names, pubchem CIDs, SMILEs (chemical structure) or any shared synonym (Kim S *et a*l. 2015). When available, the drug name from the Repurposing Hub App (Broad Institute) is shown for the user’s interface (Corsello SM et al. 2017), otherwise the main name for the compound described in PubChem data portal is shown.

# S2. Full Gene Annotation

A broad gene annotation was performed amongst all the genes available in the vulcanSpot database to improve the user experience and facilitate further interpretation. The *mygene* R package was used to obtain a summary of the biological function of the gene and alternative gene identifiers (Mark A *et al.* 2014). Genes with a oncogenic role were annotated using the Cancer Gene Census (a list of cancer genes curated by experts) from the COSMIC database (Accessed on Oct 4th 2017, Futreal PA *et al*., 2004). Genes predicted with high confidence that harbor driver variants in cancer were annotated using the list provided in Tamborero *et al.* 2013.

# S3. Defining a collection of recurrent cancer gene dependencies in cancer

The sources of evidence of cancer gene dependencies (GDs) are datasets of systematic gene loss-of-function (LoF) screenings upon a large panel of cancer cell lines (n > 400 cell lines) using RNAi or CRISPR assays (suppl. Table 1). Beyond a broad core of essential genes in the human eukaryotic cell, original studies on these datasets have shown that gene essentialities are heterogeneous across cell lines. Most of these individual dependencies are specific of the genotype and/or cancer type (i.e. biological context). Moreover, large international cancer consortiums such as the The Cancer Genome Atlas and the International Cancer Genome Consortium have shown that molecular DNA alterations on gene coding regions in cancer are of a diverse spectrum as well. However, such spectrum converges into functional impact relying on the role of the affected gene (e.g. activating or inactivating mutations of cancer genes). Thus, vulcanSpot assumes that to identify GDs associated to recurrent molecular alterations in cancer, the panel of cancer cell lines must be stratified by leveraging the impact of DNA alterations over the function of the gene affected (Gain-of-Function or Loss-of-Function) and the biological settings where these alterations take place (Pan-Cancer or the distinct cancer types). The aggregation of cell lines which harbor a common genetic alteration (e.g. a gain-of-function of ERBB2 by any missense mutations or genomic amplification) and belong to a certain cancer type (e.g. breast cancer) were compared to the background of the rest of cell lines - that do not belong to this group - in order to test for statistical associations of genotype-specific GDs.

## S3.1. Molecular stratification of cancer cell lines

DNA alterations from cancer cell lines were obtained from the CCLE data portal (<https://portals.broadinstitute.org/ccle>, Suppl. Table 1). DNA variants (point somatic mutations and copy-number alterations) on gene coding regions were transformed based on the functional impact. These DNA variants were classified by their consequence as Gain-of-Function (GoF), Loss-of-Function (LoF) and neutral/non-altered. For this purpose, cancer cell profiles of somatic point mutations and gene copy-number alterations were integrated and processed at once in a single-sample basis. Gene LoF was assigned when the gene is affected by any somatic mutation at high variant allele frequency (homozygous; VAF ≥ 0.7) whose consequence truncate the function of the gene (De_novo_Start_OutOfFrame, Frame_Shift_Del, Frame_Shift_Ins, In_Frame_Del, In_Frame_Ins, Nonsense_Mutation, Nonstop_Mutation, Splice_Site, Start_Codon_Del, Start_Codon_Ins, Stop_Codon_Del, Stop_Codon_Ins) or by a genomic deletion (CNV ≤ 0.5 gene copies). In opposite, Gene GoF was assigned when the genomic segment of DNA that contains the gene was amplified at a very high magnitude (CNV ≥ 8 gene copies) and was not affected by any truncating mutation. The particular case of somatic missense variants, which represents the most frequent point mutation in the dataset, was handle as a special case where the functional impact of the alteration relied on the zygosity of the variant and the role of the affected gene in cancer (Cancer Gene Census). In this regard, missense variants that affect oncogenes (heterozygous; VAF ≥ 0.2) were classified as GoF. Otherwise, homozygous missense variants (VAF ≥ 0.7) affecting genes with other roles in cancer (tumour suppressor genes and others) were classified as LoF.

Next, cancer cell lines which harbor a common molecular alteration with the same functional impact were grouped for statistical comparison. A Pan-Cancer group was established by aggregating all the cell lines with the same alteration regardless of the cancer type. Moreover, this group was stratified by their primary tumour site of origin to find context-specific vulnerabilities (suppl. Table 2). Both genotype-pancancer and genotype-context specific set of cell lines were subjective of a genome-wide testing for genetic dependencies.

## S3.2. Statistical testing for Genetic Dependencies (GDs)

vulcanSpot output includes inferred results from gene loss-of-function (LoF) screenings using two platforms (CRISPR and RNAi) from the Cancer Dependency Map (<https://depmap.org/>, suppl. Table 1). These datasets contain single-sample gene essentiality scores (so-called gene solutions) for around ~17,000 genes and 436-711 cell lines, for CRISPR and RNAi respectively.

For the statistical association of cancer GDs, a genome-wide Kolmogorov-Smirnov (KS) test was performed using all the genotype-context genetic alterations (obtained from section S3.1). Suppl. Figure 1 depicts the statistical test using one example. The KS was performed using the following parameters: sample size above 5 cell lines, and 10,000 permutations. This procedure tests whether there is an enrichment of the genotype-context specific cell lines as the most dependent to the function of a particular gene (the same gene that is altered, or another one) as compared to the background of the rest of cell lines. We discarded tests for GD associations with flat patterns, only retrieving genes which show a skewness distribution towards dependency (skewness ≤ -0.5). Tests for GD associations where the second gene was already affected by a LoF were omitted. Therefore, genotype-context GDs associations were established by each pair of genes using this approach (suppl. Table 3). The association takes the form “when Gene A is altered by a GoF/LoF in certain context, Gene B is essential for cell viability”. Individual scores for each GD (GD score) was established by subtracting the medians of the gene dependency scores between the cell lines that carry the genetic alteration and those without it. Genotype-context GDs with FDR < 0.25 (after multiple testing correction) were considered significant. As it is shown in suppl. Figure 2, the obtained significance after multiple testing correction was not associated to the sample size of the genetic alterations tested.

## S3.3. Correcting multi-concurrent associations to GDs

Multiple genotype-context alterations are co-occurring, thus several of these alterations are associated to the same GD. Unfortunately, these phenomena confound the biological interpretation.

In order to discern the causative players of these GDs, we assume a parsimonious cancer evolution model where cancer cells accumulate a few driver events that increase cancer fitness. Thus, these driver events are positively selected together with multiple passenger alterations that are concurrent with the driver event during the progression of the disease. These passenger alterations would be associated to the same GDs, but are unlikely the causative of the GD.

Using this simple model, we found two sources of guilty-by-association passenger alterations:

- Passenger concurrent alterations (any GoF or LoF) which are present in a subset of a larger set of cancer cell lines that harbor an oncogenic dependency (i.e. GoF of a proto-oncogene that leads to the dependency to the activated oncogene). These events are identified because are self-contained with driver events (intersection of 80%).
- Passenger concurrent alterations by copy-number variants of large segments of DNA which span a driver event. These cases are identified because they are present in the same cancer cell lines with the same type of alteration (jaccard index > 0.65) and located in the same genomic region than the driver event (minimum distance of 2Mb between genes along the genomic region).

These passenger concurrent alterations were identified and discarded from the final results afterwards.

# S4. Drug prescription amongst cancer GDs

The ultimate goal of vulcanSpot is to prioritize therapies amongst the identified GDs in the previous step. To this aim, vulcanSpot performs a comprehensive annotation of drug-gene target associations using two complementary approaches described in S4.1. and S4.2.

## S4.1. Knowledge-based drug prescription using PanDrugs

vulcanSpot interrogates druggability of GDs using the Drug Score (DScore) of direct drug-gene target associations calculated by PanDrugs v. 2018.04.30 (Piñeiro-Yáñez E et al., 2018). DScore estimates drug response and treatment suitability and takes into account: (1) drug-cancer type indication (from the FDA and clinicaltrials.gov); (2) drug clinical status (approved by the FDA, clinical trials, or preclinical); (3) gene–drug relationship (only direct targets have been considered in vulcanSpot) and (4) number of curated databases supporting gene-drug relationship. DScore has values from − 1 to 1 where negative values correspond to drug unresponsiveness and positive values to drug sensitivity.

At the time of writing, PanDrugs database included 9092 drugs, 4804 unique genes, and 43,909 direct and non-redundant gene–drug interactions. PanDrugs is publicly accessible at http://www.pandrugs.org

## S4.2. Drug repositioning of drugs whose transcriptional changes mimic the depletion of the cancer gene dependency

vulcanSpot employs drug repositioning strategy to complement and extend the known therapeutic options. During the previous approach (see S4.1 section), there are two extreme scenarios. On one hand, some actionable GDs are prescribed with several drugs that are able to directly target the cancer vulnerability, but no further biological information is provided about which compound over-performs the other therapeutic options. Therefore, additional evidences of effectiveness for certain drugs are remarkably important to prioritize these cases. On the other hand, knowledge of drug-gene targets associations is limited at present, thus many essential genes have no drug associations. Fortunately, drug repurposing could fill these gaps with the discovery of new drug-gene associations for this condition, leading to a complementary prescription of new treatments to exploit undruggable cancer vulnerabilities.

Drug repositioning was performed based on transcriptome profiling of cancer cell lines exposed to a large catalog of compounds. It seeks for compounds whose transcriptional induced-changes mimic the transcriptional effect of knocking-down the essential gene using a connectivity map approach (Lamb J *et al.*, 2006).

Additionally, vulcanSpot includes a correction of the similarity score by the proximity between the catalog of compounds to the target gene of interest in a Drug-Protein-Protein Interaction (Drug-PPI) network to promote specificity of the repurposed drugs.

### S4.2.1. Extraction of consensus cellular-specific gene expression signatures

The Library of Network-Based Cellular Signatures (LINCS) L1000 data set (Broad Institute LINCS Center for Transcriptomics (1U54HL127366)) from the Connectivity Map (CMap) project provides a large-scale catalogue of transcriptional responses to pharmacological and genetic perturbations upon a large panel of cell lines (Subramanian A *et al.,* 2017).

vulcanSpot has utilized the data corresponding to compounds (CP) and gene knock-down (KD) over the core of cell lines from the CMap, which has been tested with a large and fixed library of perturbations (suppl. Table 2). Only compounds with a known drug name were considered to create the reference dataset (n=3583 unique drugs), discarding all the small molecule compounds with no reference name from the library.

Consensus cellular-specific perturbation signatures were generated from the LINCS L1000 level 3 data (quantile log2 normalized gene expression profiles) using *limma* (v. 3.24.15, R package), by comparing the differential gene expression between treated and untreated control experiments for a given perturbation using an additive linear model to block for batch effects and individual cell lines across experiments (Custom CLUE R tools, suppl. Table 1). These are considered consensus signatures because they summarize the common transcriptional changes induced in any cell line by the perturbation including different vendors of the perturbation, replicates, different drug concentrations, measurement at different time points, and the agreement of the transcriptional effects of distinct shRNA hairpins knocking-down the same gene. In total, a reference dataset of perturbation was created for each cell line including signatures for up to 3583 compounds and 4360 gene knock-downs.

### S4.2.2. Pairwise similarity calculation between gene expression signatures of gene knock-down and compound perturbations in cancer

The Total Enrichment Score ($TES$) was calculated *(1)* by measuring the similarity between each pair of gene expression signatures derived from gene knock-down experiments and compound experiments from the LINCS L1000 project (Subramanian A *et al.,* 2017):

$TES = [ {( S}_{KD_{i} up}-S_{KD_{i} dn} ) +{( S}_{CP_{j} up}-S_{CP_{j}dn} ) ] / 2$ (1)

Where $S_{KD_{i} up}$ is the Kolmogorov-Smirnov statistic $(S)$ for the top-250 up-regulated genes by a given $KD_{i}$ gene knock-down on the ranking of differential gene expression of treated cell lines with a particular $CP_{j}$compound. $S_{KD_{i} dn}$ is the Kolmogorov-Smirnov statistic $(S)$ for the top-250 down-regulated genes by a given $KD_{i}$gene knock-down on the ranking of differential gene expression of treated cell lines with a particular $CP_{j}$ compound. Similarly, $S_{CP_{j}up}$ and $S_{CP_{j}dn}$ correspond to the same calculation for $CP_{j}$ compound but on the ranking of differential gene expression of those cell lines with a particular gene knockdown.

The $TES$ is normalized ($\tau$) (*2*) by rescaling to a $[-1, +1]$ range that indicates the proportion of times that the $CP_{j}$compound have obtained a higher value with other KD signatures (${KD}_{1..N}$) from the reference context dataset (cell lineage). The higher is the 𝜏, the more similar and specific is the similarity between ${CP}_{j}$ and ${KD}_{i}$ within the dataset.

$\tau_{{CP}_{j}{, KD}_{i}}= sign({TES}_{KD_{i},CP_{j}}) \frac{1}{(N-1)}\sum_{j=1}^{N} \left[ \left| {TES}_{KD_{i},CP_{j}} \right|>\left| {TES}_{KD_{1..N},CP_{j}} \right| \right]$ (2)

This procedure (𝜏) is very similar to that proposed by the leading team of the original LINCS L1000 dataset, but using the Total Enrichment Score ($TES$) proposed in Iorio *et al* instead of the *Weighted Connectivity Score* ($WCTS$) (Subramanian A et al. 2017).

Then, TES is normalized by the number of times that a particular compound obtains a score on the same or higher magnitude amongst the rest of gene knock-down perturbation from the database. Thus, the normalized similarity score avoids promiscuous compounds on the top of the ranking, and retrieves those that are highly specific for the KD-CP relationship.

### S4.2.3. Adjustment of the similarity score by closeness between the compound and the gene target in a contextualized Drug-Protein-Protein Interaction (Drug-PPI) network

An *ad-hoc* universal Drug-PPI network was built by appending a human Protein-Protein Interaction (PPI) network with known drug-gene target data. Protein Interaction Network Analysis (PINA) platform (Cowley M.J *et al.* 2012), which is a metadatabase that integrates PPI data from 6 public curated databases, was used to create the scaffold of the network. Only PPI supported by at least two databases and annotated as physical and direct interactions (MI:0218 and MI:0407, respectively) were retrieved for network reconstruction. Drug-gene target associations obtained from the API Restful Service from CLUE data portal ([www.clue.io](http://www.clue.io), Broad Institute) were added to the PPI network (suppl. Table 1).

For each cell line from the LINCS L1000 dataset, an individual Drug-PPi network was contextualized by removing proteins whose protein-coding genes were transcriptionally inactive in the basal gene expression profile of the cell line. For this, gene expression profiles from cancer cell lines were obtained from the CCLE data portal. The read counts from RNA-Seq were transformed into UPC values (from 0 to +1 scale; the higher is the UPC value, the more likely is that the gene is transcriptionally active) using the *SCAN.UPC* (v.2.20.0, R package) (Piccolo SR *et al.* 2013). Genes with UPC values = 0 were considered transcriptionally inactive genes.

Each contextualized Drug-PPI network was used to calculate the degree of compounds and the shortest path distance between each compound to all the genes through the PPI network. For those compounds with unknown targets, a degree of 1 and an average distance calculated from the rest of compounds to any other gene was used instead. These network properties were used to adjust the score of similarity ($\tau$) and calculate a Knock-Down~Compound Perturbation score (KDCP score) as follows:

$Prob\left( shortest distance | {CP}_{j}, {KD}_{j} \right)=\frac{1}{1000} \sum_{i=1}^{1000} {SD}_{{CP}_{j},{KD}_{i}}^{k}< {SD}_{{CP}_{j},{KD}_{i}}$

$KDCP score =\tau_{{CP}_{j}{, KD}_{i}} / \sqrt{1+Prob\left( shortest distance | {CP}_{j}, {KD}_{i} \right)}$ (3)

Where $\tau$ is the similarity score between gene expression signatures calculated in the previous section, and $Prob\left( shortest distance | {CP}_{j}, {KD}_{j} \right)$is the probability of finding a shortest distance ($SD$) between ${CP}_{j} and {KD}_{j}$amongst 1000 randomized Drug-PPI Networks preserving the degree (node connectivity) distribution of the original network.

# S5. Therapeutic prioritization

vulcanSpot final output offers a ranking of prioritized drugs to target statistically significant GDs (FDR_GD_<0.25). The ranking score is ordered decreasingly following the rational criteria:

(1) Both gene A and gene B are druggable and meet DScore ≥ 0.6 and KDCP score ≥ 0.9.

(2) Gene B is druggable meeting both DScore ≥ 0.6 and KDCP score ≥ 0.9. Gene A is undruggable.

(3) Gene B is druggable meeting either DScore ≥ 0.6 or KDCP score ≥ 0.9. Gene A is undruggable.

(4) Both gene A and gene B are undruggable.

Both of these thresholds are based on:

DScore ≥ 0.6 determines the exact cutoff that discern experimental drugs (a chemical compound with bioactivity, DScore < 0.6) from cancer drugs that are approved or under clinical trials at least (DScore ≥ 0.6) (Piñeiro-Yáñez E et al. 2018), thus showing an empirical evidence for cancer therapy.

KDCP score ≥ 0.9. corresponds to the same cutoff (tau of 0.9) recommended by the leading Connectivity Map team when using the analogous Weighted Connectivity Score (WCTS) (Subramanian A et al. 2017). It is an empirically determined cutoff based on the KDCP score distribution along the different context (supplementary figure 3), where a low kurtosis was observed and a high cutoff was define to only retrieve the highest candidates.

vulcanSpot webtool shows the top-10 compounds by default but users can expand the output and download full results table.

# S6. Description of the database

The first release of vulcanSpot database considers a feature universe of 36,078 human genes, from which 19,692 genes were altered in at least one cancer cell line from the CCLE. After filtering criteria and statistical testing, only 639 genes of these genes (genes A) were associated to GDs, with 170 GDs on average per gene A. Notably, already known cancer genes represent the 19.1% (122 out of 639) of all genes A and were associated to a greater number of GDs (364.07 on average) as compared to the non-described genes in cancer. On the other hand, a total of 7,143 genes composed all the genetic vulnerabilities (i.e. gene B). In contrast, a very small fraction of the genes B were currently classified as cancer driver genes (5.5%, 394 out of 7,143), suggesting that the vast majority of cancer genetic vulnerabilities are actionable through genes (genes B) with still unknown cancer role.

# S7. Recall of canonical cancer gene dependencies

vulcanSpot was able to identify some of known gene dependencies already reported in the literature. For instance, several oncogenic addiction were found essential to maintain cancer cell fitness in those cells with activating mutations in oncogenes, including self-dependency for the oncogenic form of BRAF (melanoma), ERBB2 (breast cancer), KRAS (pancreatic cancer), MYCN (neuroblastoma), CTNNB1 (large intestine - gastrointestinal cancer), or downstream signaling effectors NFKB1 (KRAS mutant, haematological malignancies) and PIK3CA (ERBB2 mutant, breast cancer) (Pagliarini et al. 2015). Synthetic lethals already described in the literature were confirmed such as CDKN2A - CDK4/6 (pan-cancer), RB1 - SKP2 (pan-cancer), SMARCA4 - SMARCA2 (pan-cancer) (Brunen et al. 2017).

Known effective targeted therapies among these cancer vulnerabilities were also validated using using cell lines drug sensitivity data (IC50) from GDSC. These validated drug prescriptions were ranked on top positions by vulcanspot. Dabrafenib or vemurafenib for the treatment of BRAF mutant melanoma tumours; CP-724714 (or afatinib, neratinib) for breast tumours with gain-of-function on ERBB2; pictilisib for breast cancer with gain-of-function in PIK3CA, and palbociclib (CDK4/6 inhibitor) for any tumour with the tumour suppressor gene CDKN2A impaired (supplementary figure 4).

# S8. Validation of best candidates of therapeutic vulnerabilities using CTRP

GeneA-drug pairs with a KDCP score greater than 0.9 (n=402) or a Pandrugs score greater than 0.6 (n=669) in pan-cancer context were considered. These therapeutic vulnerabilities are those defined as best candidates by vulcanSpot. Cancer cell lines drug response data was downloaded from the Cancer Therapeutics Response Portal (CTRP, https://portals.broadinstitute.org/ctrp/) and cancer cell line mutation calls (coding region) were downloaded from DepMap 19Q1 release (https://depmap.org/portal/download/). Only pan-cancer was considered because splitting into cancer types limits the sample size. All cell lines in which the drug was tested were selected. Cell lines were then separated in two groups: those with mutations in gene A and those without mutations in gene A. Only Missense mutations, nonsense mutations, frameshift insertions and frameshift deletions were considered. A Wilcoxon signed-rank test was used to probe if statistically significant differences were observed in drug response, using the Area under the curve as a measure.

In order to validate the relationship between essentiality and drug response, GeneB-drug pairs with a KDCP score greater than 0.9 (n=5419) or a Pandrugs score greater than 0.6 (n=203) in pan-cancer context were considered. Cancer cell lines drug response data was downloaded from the Cancer Therapeutics Response Portal (CTRP, https://portals.broadinstitute.org/ctrp/) and gene essentiality scores from whole-genome CRISPR-Cas9 assays were downloaded from DepMap 19Q1 release (https://depmap.org/portal/download/). Only pan-cancer was considered because splitting into cancer types limits the sample size. All cell lines in which the drug was tested and in which CRISPR was assayed were selected. The Spearman correlation coefficient was used to check if a statistically significant correlation existed between the essentiality of gene B (as measured by the CERES score) and drug response, using the Area under the curve as a measure.

# REFERENCES

Kim S, Thiessen PA, Bolton EE, Bryant SH. PUG-SOAP and PUG-REST: web services for programmatic access to chemical information in PubChem. Nucleic Acids Res 2015 Jul 1;43(W1):W605-11. Epub 2015 Apr 30 [PubMed PMID: 25934803] doi: 10.1093/nar/gkv396.

Corsello SM, Bittker JA, Liu Z, Gould J, McCarren P, Hirschman JE, Johnston SE, Vrcic A, Wong B, Khan M, Asiedu J, Narayan R, Mader CC, Subramanian A, Golub TR. The Drug Repurposing Hub: a next-generation drug library and information resource. Nature Medicine. 23, 405–408 (2017)

Mark A, Thompson R, Afrasiabi C, Wu C (2014). mygene: Access MyGene.Info_ services. R package version 1.16.0.

Futreal PA et al. (2004). A census of human cancer genes. Nature reviews. Cancer 2004;4;3;177-83

Tamborero D, Gonzalez-Perez A, Perez-Llamas C, Deu-Pons J, Kandoth C, Reimand J, Lawrence MS, Getz G, Bader GD, Ding L, Lopez-Bigas N. Comprehensive identification of mutational cancer driver genes across 12 tumor types. Sci Rep. 2013 Oct 2;3:2650

Lamb J, Crawford ED, Peck D, Modell JW, Blat IC, Wrobel MJ, Lerner J, Brunet JP, Subramanian A, Ross KN, Reich M, Hieronymus H, Wei G, Armstrong SA, Haggarty SJ, Clemons PA, Wei R, Carr SA, Lander ES, Golub TR. The Connectivity Map: using gene-expression signatures to connect small molecules, genes, and disease. Science. 2006 Sep 29;313(5795):1929-35.

Piñeiro-Yáñez E et al. (2018). PanDrugs: a novel method to prioritize anticancer drug treatments according to individual genomic data. Genome Med. 10(1):41.

Subramanian A et al. (2017). A Next Generation Connectivity Map: L1000 Platform and the first 1,000,000 Profiles. Cell, 171(6):1437-1452.

Cowley M.J., Pinese M., Kassahn K.S., Waddell N., Pearson J.V., Grimmond S.M., Biankin A.V., Hautaniemi S. and Wu, J. (2012) PINA v2.0: mining interactome modules. Nucleic Acids Res, 40, D862-865

Piccolo SR, Withers MR, Francis OE, Bild AH, Johnson WE (2013). “Multi-platform single-sample estimates of transcriptional activation.” Proceedings of the National Academy of Sciences of the United States of America, 110(44), 17778-17783.

Pagliarini R, Shao W, Sellers WR. Oncogene addiction: pathways of therapeutic response, resistance, and road maps toward a cure. EMBO Rep. 2015 Mar; 16(3): 280–296.

Brunen D, Bernards R. Drug therapy: Exploiting synthetic lethality to improve cancer therapy. Nat Rev Clin Oncol. 2017 Jun;14(6):331-332.

# Supplementary Tables and Figures

**Supplementary Table 1.** Resources integrated in vulcanSpot for the identification and drug prescription of therapeutic cancer genetic vulnerabilities.

| **RESOURCE** | **SOURCE** | **DESCRIPTION** | **IDENTIFIER** |
| --- | --- | --- | --- |
| Gene Expression Profiles | <https://portals.broadinstitute.org/ccle/data> | CCLE RNAseq gene expression data (read count). | CCLE_RNAseq_081117.reads.gct |
| Point somatic mutations | <https://portals.broadinstitute.org/ccle/data> | CCLE Merged mutation calls (coding region, germline filtered). | ccle2maf_081117.txt |
| Gene Copy-number Alterations | <https://portals.broadinstitute.org/ccle/data> | CCLE Copy-number values per gene. | CCLE_copynumber_byGene_2013-12-03.txt |
| Combined RNAi | <https://depmap.org> | Final dataset from [(McFarland et al. 2018)](https://paperpile.com/c/rxm9NW/fiVE) using DEMETER2 algorithm. | portal-RNAi_merged-2018-05-10.csv |
| DepMap CRISPR (Avana) 18Q2 | <https://depmap.org> | Final dataset from [(Meyers et al. 2017)](https://paperpile.com/c/rxm9NW/k29q) using CERES algorithm. | portal-Avana-2018-05-10.csv |
| Cancer Driver list | Tamborero *et al.* (2013). Sci Reports. Supplementary Table 2. | List of High Confidence Drivers and Confidence Drivers with their signals of positive  selection annotated in Tamborero *et al.,* 2013. | srep02650-s3.csv |
| Cancer Gene Census | COSMIC data portal | List of Oncogenes & Tumour Suppressor Genes (Accessed on the 4th Oct 2017). | Census_allWed_Oct_4_09-53-19_2017.tsv |
| Protein-Protein interaction Network | <http://omics.bjcancer.org/pina/interactome.stat.do> | Integration of protein-protein interaction in human data from six public curated databases: IntAct, BioGRID, MINT, DIP, HPRD, MIPS/MPact. No. binary interactions: 166,776; No. complexes: 5211. | Homo sapiens-20140521.tsv (2014) |
| Drug-Target regulatory Network | <https://clue.io/api> | Database of drug-target direct genes relationships from Corsello SM *et al*. 2017. | Queried on the 17th August 2017 using the "Custom CLUE R tools" |
| PanDrugs | <http://www.pandrugs.org> | Pharmacological data and drug annotations collected from 24 databases. | version 2018.04.30 |
| LINCS L1000 v1 dataset | http://lincscloud.org (website deprecated, now available as two separated datasets at Gene Expression Omnibus (GEO): GSE92742 and GSE70138) | Binary GCT matrix with transcriptomic profiles for the experiments of LINCS L1000 Phase I and Phase II. | q2norm_n1328098x22268.gctx (level 3) [md5: efe18d9e0816b6ffc3e21cf0a98455a4] |
| Custom CLUE R tools | <https://github.com/jperales/CLUE> | Custom package of R functions to extract gene expression signatures from LINCS L1000 dataset. | SHA: bf891998c064014a59def4d98034710ffd802021 |
| CTRPv2 drug sensitivity data | <https://portals.broadinstitute.org/ctrp/> | area-under-concentration-response curve (AUC) sensitivity scores for each cancer cell line and each compound | v20.data.curves_post_qc.txt |

**Supplementary Table 2.** Set of cell lines included by context

| **Context** | **Core of cell lines from LINCS L1000** |
| --- | --- |
| ADRENAL_CORTEX |  |
| AUTONOMIC_GANGLIA |  |
| BILIARY_TRACT |  |
| BONE |  |
| BREAST | BT20,HS578T,MCF7,MDAMB231,SKBR3 |
| CENTRAL_NERVOUS_SYSTEM |  |
| CERVIX |  |
| ENDOMETRIUM |  |
| HAEMATOPOIETIC_AND_LYMPHOID_TISSUE |  |
| KIDNEY |  |
| LARGE_INTESTINE | CL34,HCT116,HT115,HT29,LOVO,MDST8,NCIH508,NCIH716,RKO,SNU1040,SNUC4,SNUC5,SW480,SW620,SW948 |
| LIVER | HEPG2,HUH7 |
| LUNG | A549,CORL23,DV90,HCC15,HCC515,NCIH1694,NCIH1836,NCIH2073,NCIH596,SKLU1,T3M10 |
| OESOPHAGUS |  |
| OVARY |  |
| PANCREAS |  |
| PLACENTA |  |
| PLEURA |  |
| PROSTATE | PC3,VCAP |
| SALIVARY_GLAND |  |
| SKIN | A375,SKMEL1,SKMEL28 |
| SMALL_INTESTINE |  |
| SOFT_TISSUE |  |
| STOMACH |  |
| TESTIS |  |
| THYROID |  |
| UPPER_AERODIGESTIVE_TRACT |  |
| URINARY_TRACT |  |
| VULVA |  |
| PANCANCER | A375,A549,A673,AGS,BT20,CL34,CORL23,COV644,DV90,EFO27,HA1E,HCC15,HCC515,HCT116,HEC108,HEKTE,HEPG2,HL60,HS578T,HT115,HT29,HUH7,JHUEM2,JURKAT,LOVO,MCF7,MDAMB231,MDST8,NCIH1694,NCIH1836,NCIH2073,NCIH508,NCIH596,NCIH716,NOMO1,OV7,PC3,PL21,RKO,RMGI,RMUGS,SKBR3,SKLU1,SKM1,SKMEL1,SKMEL28,SNGM,SNU1040,SNUC4,SNUC5,SW480,SW620,SW948,T3M10,THP1,TYKNU,U2OS,U937,VCAP,WSUDLCL2 |

**Supplementary table 3.** Number of features for statistical testing in Gene Dependencies (GDs). Note that for all the genetic alterations there was enough sample size to perform the statistical test.

| Dataset | Context-specific genetic alterations | GDs on Genes B | Total comparisons |
| --- | --- | --- | --- |
| RNAi | 6,124 | 3,367 | 15,605,722 |
| CRISPR | 3,155 | 4,862 | 15,339,409 |

**Supplementary Figure 1.** Depiction of the identification of cancer gene dependencies (Step 1 from Figure 1). The molecular profiles from cancer cell lines were obtained from the CCLE data portal (mutational status) and Cancer Dependency Map (gene dependency). Cell lines were stratified by the functional impact of genetic alterations (GoF/LoF) affecting Genes A (section S3.1). Then, a Kolmogorov-Smirnov (KS) test is performed using every genetic alteration to test whether there is an enrichment of cell lines which carry the alteration as the most dependent to the function of a second gene. The test is performed using all the cancer cell lines from the panel as a background for the comparison. For instance, it will test whether cell lines with SMARCA4 impaired by LoF mutations are enriched as those more dependant to the function of a second gene in a genome-wide fashion. Eventually, the algorithm will find statistical significance when SMARCA4-impaired cell lines are more dependent to SMARCA2 as compared to the rest of cell lines (SMARCA4 wild-type).


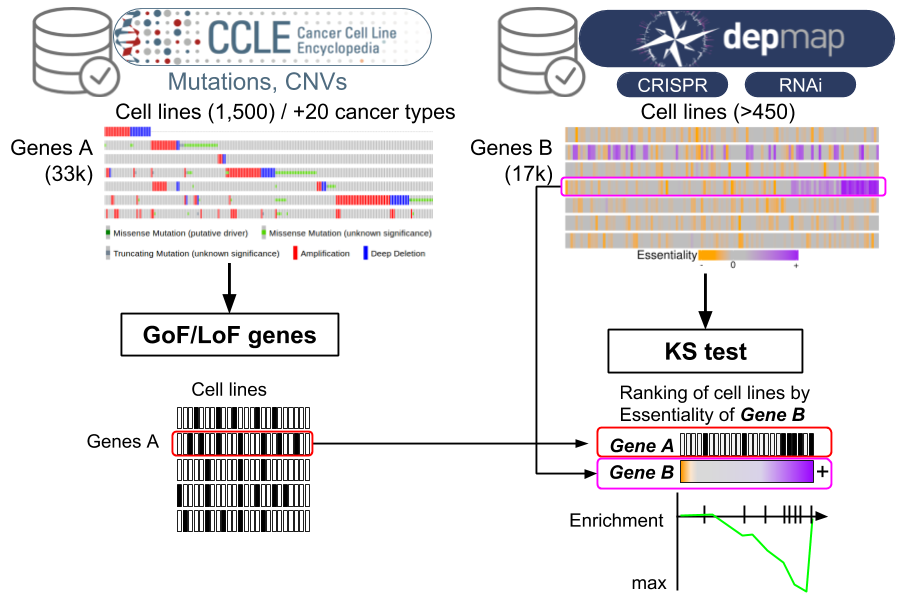


**Supplementary Figure 2.** Volcano plot of the direction of enrichment versus the significance from the identification of gene dependencies (Step 1 from Figure 1). Only a set of 500,000 randomly selected tested associations are shown for clarity. The two datasets of genome-wide loss-of-function screenings are shown. Size of the points indicate the sample size (i.e. the number of cell lines with the mutation). The two horizontal lines corresponds to FDR=0.05 and FDR=0.25.


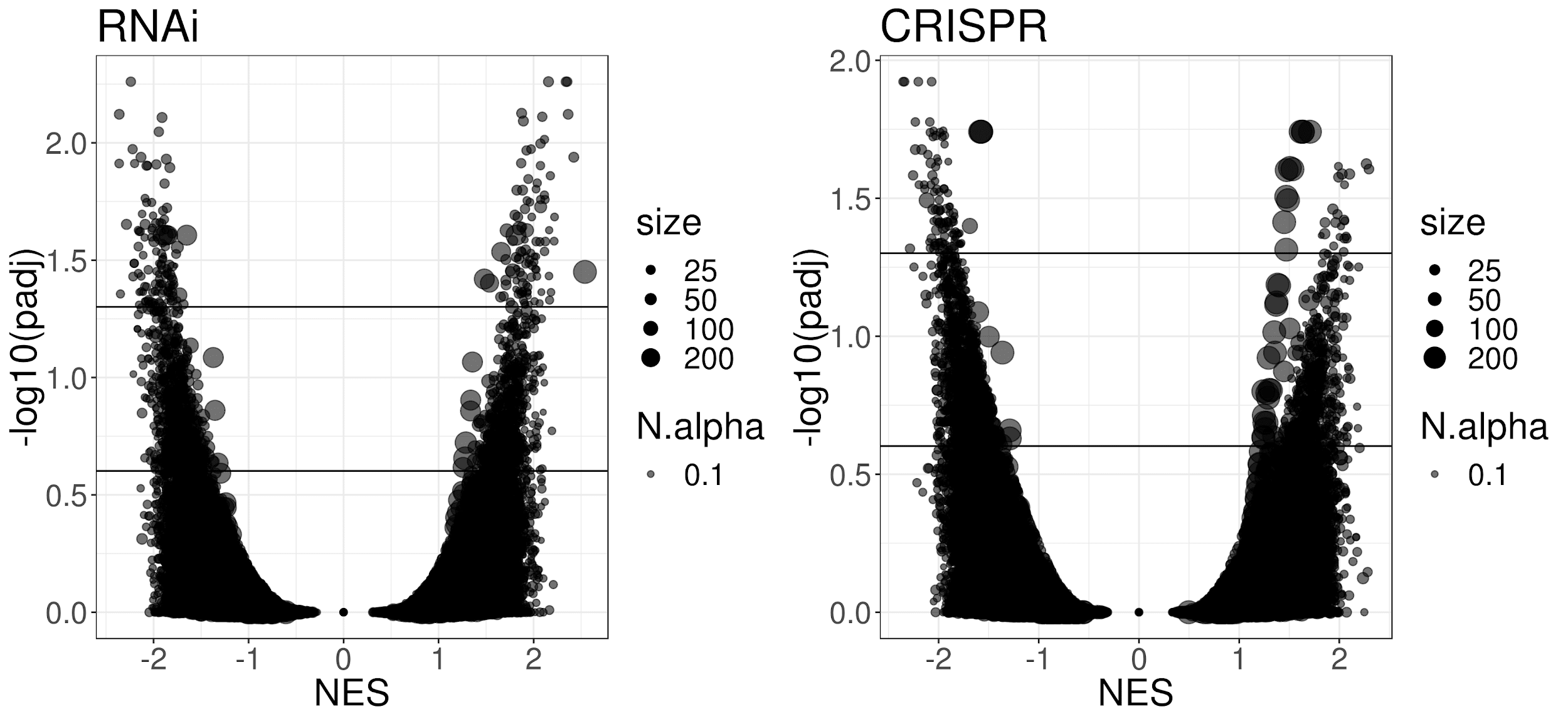


**Supplementary Figure 3.** KDCP score distribution across cancer contexts. The vertical line indicates the arbitrary threshold at KDCP score = 0.90 for ranking best candidates.


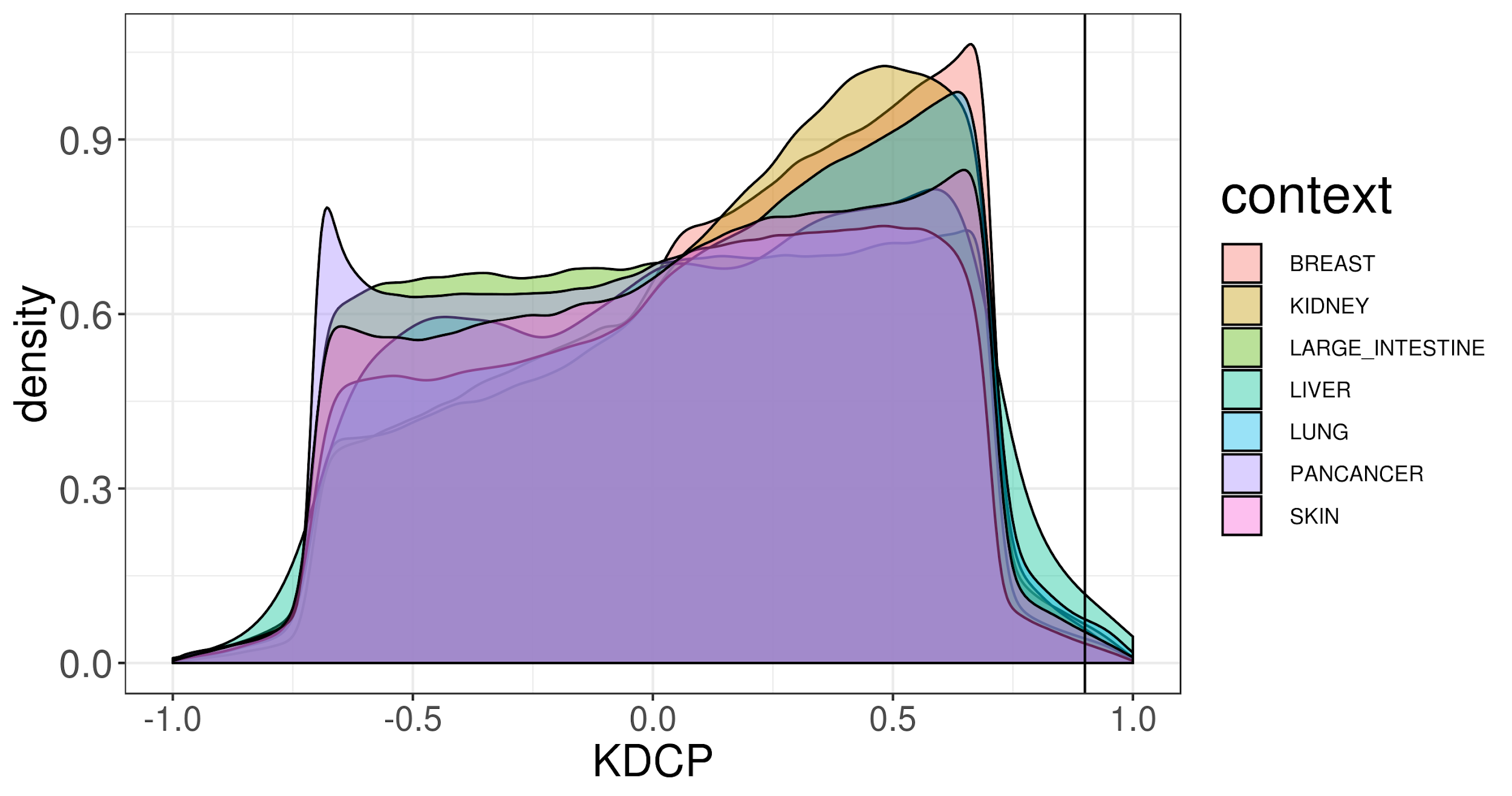


**Supplementary Figure 4.** Examples of therapies to target known genetic dependencies proposed by vulcanspot were validated using cell lines drug sensitivity data (IC50) from GDSC.


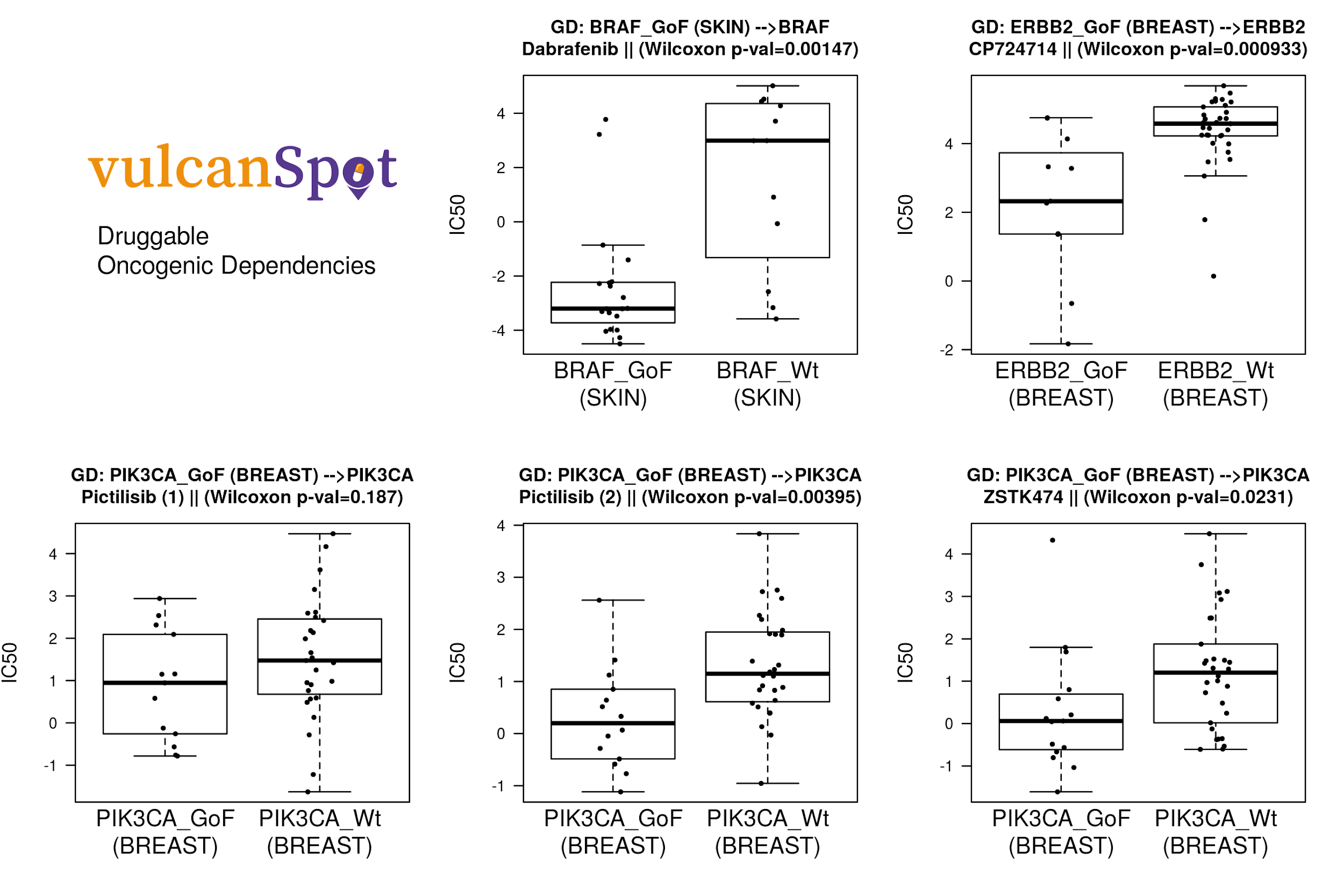

Supplement: btz465_Supplementary_Data [file btz465_supplementary_data.docx]
